# Roles and action mechanisms of bile acid-induced gastric intestinal metaplasia: a review

**DOI:** 10.1038/s41420-022-00962-1

**Published:** 2022-04-04

**Authors:** Qijin He, Limin Liu, Jingge Wei, Jiaying Jiang, Zheng Rong, Xin Chen, Jingwen Zhao, Kui Jiang

**Affiliations:** Department of Gastroenterology and Hepatology, Tianjin Medical University General Hospital, Tianjin Institute of Digestive Diseases, Tianjin Key Laboratory of Digestive Diseases, No. 154 Anshan Road, Tianjin, 300052 China

**Keywords:** Oncogenesis, Gastrointestinal diseases

## Abstract

Gastric intestinal metaplasia (IM) is a precancerous lesion that increases the risk of subsequent gastric cancer (GC) development. Therefore, the mechanism of IM has been the focus of basic and clinical research. Helicobacter pylori (H. pylori) infection has been recognized as the main pathogenesis of gastric IM. However, more and more studies have shown that chronic inflammation of gastric mucosa caused by bile reflux is the key pathogenic factor of gastric IM. Bile reflux activates the expression of IM biomarkers via the bile acid receptor. In addition, microRNAs, exosomes, and epigenetics are also involved in the occurrence and development of bile acid-induced gastric IM. Currently, the relevant research is still very few. The molecular mechanism of the phenotypic transformation of gastrointestinal epithelial cells induced by bile acids has not been fully understood. This article mainly reviews the physiology and pathology of bile acid, mechanism of gastric IM induced by bile acid, bile acid receptors, and so on, in order to provide reference for further research.

## Facts

- Gastric intestinal metaplasia (IM) is a precancerous lesion of gastric cancer (GC), and early diagnosis and treatment is important.
- Accumulative evidence has revealed that bile reflux is associated with gastric IM and even carcinoma.
- Bile reflux into the stomach can not only directly stimulate the gastric mucosal barrier, but also regulate multiple downstream pathways to induce chronic inflammation of the gastric mucosa.

## Open questions

- What is the action mechanism of bile acid-induced gastric IM?
- Is gastric IM reversible?

## Introduction

**Fig. 1:**
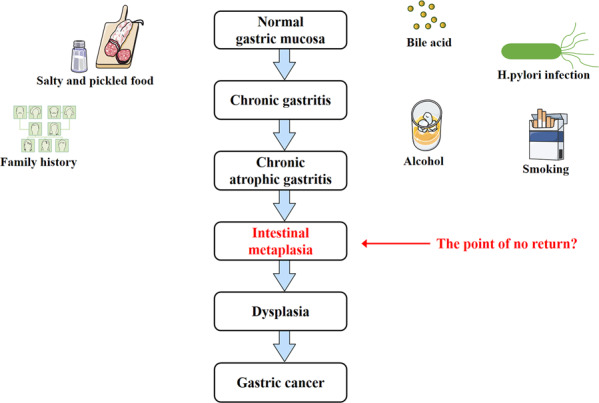
GC development following the Correa’s cancer cascade. Gastric IM is the key point for the development of GC. Whether it can be reversed is a new idea for the treatment of GC.

Gastric cancer (GC) is a common malignant tumor of the digestive system in the world, which ranks fifth in incidence and the fourth leading cause of cancer-related death [1]. Although the treatment has advanced greatly in recent decades, the prognosis for GC patients is still poor, with five-year overall survival rates ranging from 20% to 40%, mainly due to the rapid progress of local recurrences and metastasis [2]. Gastric adenocarcinoma is the most common form of GC, of which there are 2 histologic subtypes: intestinal type and diffuse type. It is generally believed that GC development, especially the intestinal type of noncardia GC, follows the Correa’s cancer cascade—a successive progression from chronic nonatrophic gastritis, by way of atrophic gastritis and intestinal metaplasia (IM), to dysplasia [3] (Fig. 1). A study in Japan demonstrated that the presence of gastric IM was the only criteria associated with the development of intestinal-type GC [4]. The relative risk of developing GC is 10 times higher for individuals with IM than for those healthy individuals [5]. Therefore, monitoring gastric IM and reversing it is an important idea to stop the development of GC. Identifying its pathogenesis and taking proper measures may become the key to GC prevention. Increasing evidence has demonstrated that bile reflux is thought to be associated with atrophic gastritis, IM, dysplasia, and even carcinogenesis [6]. However, the mechanism of bile acid-induced gastric IM in the stomach is not clear and needs further research.

## Definition, histologic subtyping, and cancer risk of gastric IM

### Definition

Gastric IM, a precancerous histopathological change, is defined as the replacement of gastric columnar cells by cells of intestinal morphology characterized by the presence of mucin-containing goblet, Paneth and absorptive cells, resulting in normal gastric mucosal epithelium and the surrounding glands are replaced by intestinal epithelium and glands [7]. Helicobacter pylori (H. pylori) infection is the most important risk factor for gastric atrophy and intestinal metaplasia [8]. In addition, male gender, old age, ethnicity, dietary factors, such as smoked food, high salt intake, pickled vegetables and nitrated meat, bile reflux, smoking, and family history are also associated with gastric IM [9, 10].

### Histologic subtyping

Most gastric IM is divided into complete IM and incomplete IM based on hematoxylin and eosin staining. Occasionally, mixed characteristics of complete and incomplete types are observed [11]. The complete type resembles small intestinal epithelium comprising mature absorptive columnar cells, goblet cells, and Paneth cells, with a brush border given by large numbers of apical microvilli. While the incomplete type displays goblet cells of variable size and intervening columnar mucin-secreting cells without a brush border [11]. Another classification system was suggested by pathologists Filipe and Jass based on morphology and classic mucin staining, which divided the gastric IM into three types: type I corresponds to complete gastric IM and types II and III are subclassifications of incomplete gastric IM [12].

### Risk of developing cancer

Liming Shao et al. systematically assessed the risk of GC in patients with gastric IM through 21 studies, which showed that incomplete IM but not complete IM was significantly associated with a higher risk of GC [13]. Incomplete IM, considered the most advanced stage of IM, is a valid marker for identifying subjects at high risk of GC [14]. Besides, it appeared that the risk of GC was higher among patients with IM in the corpus than those with IM in the antrum only [15, 16]. In clinical practice, the analysis of gastric IM subtyping using a small number of biopsies for histopathological examination is useful for monitoring the risk of GC.

## The physiology and pathology of bile acid

**Fig. 2:**
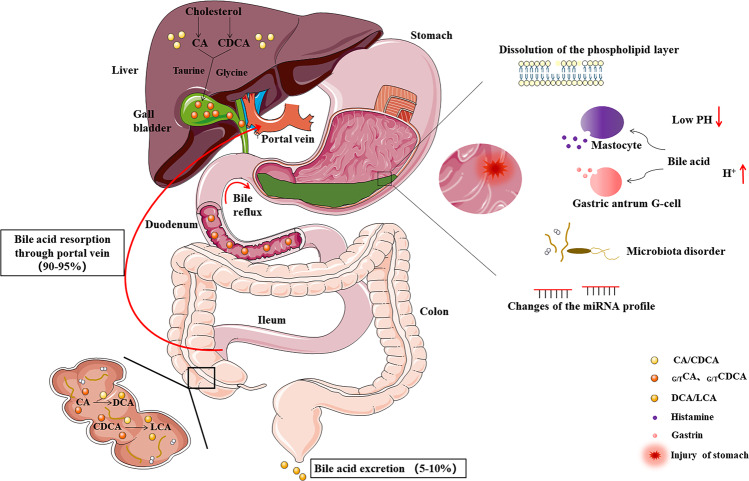
Bile acid circulation and bile reflux. Primary bile acids (e.g., CA and CDCA) are synthesized by cholesterol mediated. They conjugated with glycine or taurine (G/T), and stored in the gallbladder. The majority (90–95%) of bile acid secreted into the small intestine are reabsorbed in the terminal ileum through portal vein and circulate back to the liver. Gut microbiota contributes to the conversion of CA and CDCA into secondary bile acids through dehydroxylation (e.g., DCA and LCA), which are mostly excreted from feces (5–10% of bile acid). Bile reflux to the stomach can induce mucosal dysfunction through some factors, such as dissolution of the phospholipid layer, low pH, mastocyte-secreting histamine, gastric antrum G-cell-secreting gastrin, microbiota disorder, and changes of the miRNA profile.

Bile acids, a 24-carbon derivative of cholanic acid in structure, primarily synthesized in the liver, are the major components of bile. Primary bile acids of cholic acid (CA) and chenodeoxycholic acid (CDCA) are synthesized from cholesterol in hepatocytes and conjugated with glycine or taurine, secreted into the bile [17]. Postprandial contraction of the gallbladder drains bile acids into the intestine, and conjugated primary bile acids are stripped of glycine and taurine in the terminal ileum and colon, mostly reabsorbed into the blood to the liver [18]. A proportion is not reabsorbed and reaches more distal parts where gut bacteria can dehydroxylate the primary bile acids, forming the secondary bile acids lithocholic acid (LCA), deoxycholic acid (DCA), and their conjugated types, absorbed by passive diffusion in the colon or excreted in stool [19]. The human bile acid pool is 2–4 g, and cycles multiple times daily for a total of up to 30 g/day absorbed, with less than 10% lost in feces [20] (Fig. 2).

Bile reflux, also known as duodenogastric reflux, refers to the backflow of duodenal contents, including bile, pancreatic juice, and duodenal juice, into the stomach [21]. There are many clinical methods to diagnose bile reflux, mainly including endoscopy with histological examination, intragastric pH monitoring, radionuclide scanning, gastric aspiration, and bile detection with special probes. The normal gastric mucosal epithelium is covered with a mucous gel layer and a bicarbonate layer, which blocks the contact of harmful components in the stomach and mucosa. Under normal circumstances, primary bile acids formed in the liver are secreted into the intestine lumen, where they are modified by the intestinal bacteria to produce secondary bile acids [22]. When bile reflux occurs, secondary bile acids and free bile acids are regurgitated into the stomach. Lecithin from bile acids and bile salts is converted to lysolecithin by the action of phospholipase A, which dissolves the phospholipid layer of the epithelial cell membrane of the gastric mucosa, resulting in increased cell permeability. Bile acids inhibit the activity of NO enzyme and the sodium hydrogen exchange of cells, leading to intracellular DNA damage, apoptosis, and mutation. Bile acids can also promote the reverse diffusion of H^+^ and stimulate mast cells to release histamine, so as to stimulate gastric acid secretion and further aggravate gastric mucosal injury [23, 24]. Under low-pH conditions, bile acids are converted to nonionic forms, making them more cytotoxic, more likely to penetrate cell membranes, and damage tight intercellular junctions [25]. In addition, bile reflux can stimulate gastrin secretion from G cells, which promotes gastric acid secretion and inhibits pyloric sphincter contraction, further promoting bile reflux and forming a vicious circle. Reflux of alkaline substances from bile into the stomach can cause flora displacement, thus aggravating the inflammatory response of the gastric mucosa [26]. Recent studies have shown that acidic bile acids in gastric juice after successful Hp eradication can also induce mucosal dysfunction with changes in the microRNA (miRNA) profile, which might drive the development of gastric carcinogenesis [27] (Fig. 2).

## Mechanism of gastric IM induced by bile acid

IM is considered the point of no return in the GC. Investigating bile acid-induced IM is important and necessary for the prevention of GC. Matsuhisa et al. found that high concentrations of bile acid appeared to be associated with an elevated risk of IM, regardless of H. pylori infection [28]. Another study demonstrated that the incidence of GC was at a high rate of 41% in a rat model of bile reflux [27]. In addition, a retrospective cohort study indicated that high concentrations of bile acids in the stomach were associated with a higher incidence of GC [29]. Recent studies have shown that IM biomarkers, microRNAs (miRNAs), exosomes, and epigenetic modifications are associated with gastric IM.

### IM biomarkers

Caudal-related homeobox transcription-factor 2 (CDX2), an intestine-specific nuclear transcription factor, plays a critical role in directing intestinal development, differentiation, and maintenance of the intestinal phenotype [14]. Studies have shown that CDX2 is expressed in the intestinal metaplasia of the stomach, while it is not expressed in the normal gastric mucosa [30]. Furthermore, CDX2 transgenic mice developed IM in the stomach with the induction of mucin 2 (MUC2), while knockdown of CDX2 resulted in intestinal malformation and inactivation of the expression of IM-related immunohistochemical marker, suggesting that ectopically expressed CDX2 may play a key role in GC via induction of IM [31].

The transcription-factor sex-determining region Y box2 (SOX2) plays a pivotal role in the regulation of normal gastric phenotype [32, 33]. For example, SOX2 gradually decreased expression in the process of gastric mucosa developing into IM, but there is no expression of SOX2 in small intestinal tissues and large intestinal tissues [34, 35]. The abnormal loss of SOX2 expression is highly correlated with IM of gastric and esophageal mucosa, which can be used as a molecular marker to detect the occurrence of IM [32, 36]. Moreover, SOX2 is negatively correlated with the expression of intestinal-specific molecules such as CDX1 and CDX2 in normal gastric epithelium, atrophic gastritis, and intestinal metaplasia tissues [34, 35]. Yuan et al. found that bile acids induced the expression of miR-21, which could inhibit the expression of SOX2 by directly binding its 3′-UTR and abrogate its suppression on the transcriptional activity of CDX2, thus leading to IM [37].

Hepatocyte nuclear factor-4 alpha (HNF4α) is a ligand-activated nuclear transcription factor, which is widely associated with the transcriptional regulation of hepatocyte genes and plays a role in regulating gene expression relating to drug metabolism, lipid metabolism, cell proliferation, and inflammation [38–40]. In adults, HNF4α is expressed in the colon and small intestine, not in the gastric mucosa, but HNF4α mediated by the P1 promoter can be observed in gastric IM tissues [41]. Zhen et al. found that bile reflux activated the TGR5–ERK1/2 pathway after induction of HNF4α expression, thus upregulating the expression of CDX2 [42]. Wang et al. found that bile acids promoted the development of gastric IM through HNF4a/HDCA6/CDX2 pathway in vivo and in vitro [43].

In addition, IM biomarkers also include mucin 2 (MUC2), Kruppel-like factor 4 (KLF4), villin-1, and cadherin 17, which are downstream of CDX2. Studies have shown that the expression of them is elevated in gastric epithelial cells exposed to bile acids, leading to the genesis of intestinal metaplasia [37, 43].

### MiRNAs

MiRNA, a class of small endogenous noncoding RNA molecules, leads to mRNA degradation or translational inhibition of specific target genes by base-pairing to their mRNAs [44]. They can participate in a series of physiological and pathological processes, including development, differentiation, cell proliferation, apoptosis, organogenesis, and homeostasis [45]. Within diverse cancer types, the expression of miRNA is substantially different compared with their normal tissue [46]. Li et al. found that bile acid-stimulated IM induced the upregulation of miR-92a-1–5p [47]. Wang et al. found that DCA inhibited miR-1 in gastric cells to induce high expression of HDAC6 and HNF4α, thereby inhibiting the expression of downstream IM markers [43]. Further studies are needed to confirm the role of miRNA in gastric IM.

### Exosomes

Exosomes, a type of membrane vesicles with a diameter of approximately 40–100 nm [48], contain multiple biological molecules, such as proteins, lipids, and mRNAs, and play an important role in regulating tumor development, metastasis, and drug resistance [49, 50]. Xu et al. demonstrated that DCA-activated macrophages could secrete exosomes to carry miR-30a-5 to gastric epithelial cells, thereby promoting gastric IM by targeting FOXD1 [51]. It was found that IM might arise from spasmolytic polypeptide-expressing metaplasia (SPEM) [52]. DCA could promote SPEM by macrophage-derived exosomes [53]. Further studies are needed to explore the role of exosomes in gastric IM.

### Epigenetics

Epigenetics is defined as the study of chemical modifications of DNA and histone proteins that alter the structure of chromatin without changes to the underlying nucleotide sequence. N6 methyladenine (m6A) is the most common modification of mRNA in mammals [54]. AlkB homolog 5 (ALKBH5) is one of the currently discovered m6A demethylases, which plays an important role in the dynamic regulation of m6A [55]. Ben et al. demonstrated that ALKBH5 activated CDX2 by targeting the ZNF333/CYLD axis and activating NF-kappaB (NF-κB) signaling [56]. Therefore, targeting ALKBH5 and ZNF333 may be an effective preventive and therapeutic strategy for GIM in patients with bile reflux. Transcription-factor Dickkopf (Dkk) family is a specific antagonist of the Wnt signaling pathway, which participates in many developmental processes of embryo formation and plays an important role in maintaining adult tissue homeostasis [57, 58]. Studies have shown that enhanced expression of DKK1 has been observed in various cancers, which promotes tumor cell proliferation, invasion, and migration [59]. Lu et al. found that DKK1 was epigenetically downregulated by promoter methylation, thereby inhibiting bile acid-induced gastric IM [60].

## Bile acid receptors

Bile acid receptors, mainly including G-protein-coupled receptor 5 (TGR5), farnesoid X receptor (FXR), pregnane X receptor (PXR), constitutive androstane receptor (CAR), and vitamin-D receptor (VDR), regulate bile acid metabolism, glucose utilization, fatty acid synthesis and oxidation, energy-homeostasis balance, immune-cell function, nerve activity, and other functions [61, 62]. In gastric IM, FXR and TGR5 are widely studied. Bile acids, acting as signaling molecules, regulate downstream signal-transduction pathways by activating the nuclear receptor FXR or the plasma-membrane receptor TGR5 (Table 1).

**Table 1:** The articles on the bile acid-induced gastric IM via receptors of FXR and TGR5.

Bile acid	Receptor	Subjects	Regulatory mechanism	References
CDCA	FXR	cell lines	FXR may cause the ectopic expression of CDX2 and MUC2 in the gastric mucosa, and that CDCA may be a potent activator of the FXR in the stomach.	[69]
DCA	FXR	cell lines	DCA is capable of modulating the expression of CDX2 and the downstream MUC2 via the nuclear receptor FXR-NF-κB activity in normal gastric epithelial cells.	[70]
CDCA	FXR	tissues, cell lines	The activation of FXR and sequential direct transcriptional induction of SHP were involved in the expression of CDX2 induced by bile acid in gastric IM lesions.	[71]
DCA, CDCA	FXR	tissues, animals, cell lines	Bile acids may activate the FXR/NF-κB signaling pathway, thereby upregulating CDX2 and MUC2 expression in normal gastric epithelial cells.	[108]
CDCA	FXR	tissues, cell lines	Bile acid induces upregulation of miR-92a-1–5p through the activation of FXR, leading to a decrease of FOXD1 and a continuous activation of NF-κB pathway, which in turn promotes the transcription of CDX2 and intestinal differentiation.	[47]
DCA	FXR	tissues, cell lines	Under the stimulation of bile acids, enhanced FXR gene expression increases the expression of SNAI2, which in turn decreases the inhibitory effect of miR-1 on HDAC6 and HNF4α expression. Upregulated HDAC6 and HNF4α interact with each other, which further promotes upregulation of CDX2, KLF4 and MUC2, thereby promoting gastric IM development.	[109]
DCA	TGR5	tissues, cell lines	BAs treatment could activate TGR5–ERK1/2 pathway following induction of HNF4α expression, which further promoted metaplasia markers expression through direct regulation of KLF4 and CDX2.	[42]

### FXR

The bile acid-activated nuclear receptor FXR, mainly expressed in the liver and intestine, is a key regulator of signaling pathways and cellular functions, such as bile acid homeostasis, lipid metabolism, and glucose metabolism [63, 64]. Many studies in vitro have shown a rank order for the ability of bile acids to activate FXR:CDCA > DCA > LCA > CA, while this order had differences in mouse [65, 66]. It is generally recognized that unconjugated bile acids have greater potential to activate FXR than conjugated bile acids [67]. A previous study has reported that high expression levels of FXR are associated with the gastric IM formation [68]. Xu et al. first reported that CDCA stimulating the normal gastric epithelial cells of the rat upregulated the expression of CDX2 and MUC2 by activating FXR [69]. Subsequently, Li et al. revealed that DCA activated the FXR/NF-kappaB signaling pathway, thereby upregulating CDX2 and MUC2 expression in normal gastric epithelial cells [70]. Further, Zhou H et al. found that FXR and CDX2 were concomiantly overexpressed and were positively correlated in IM tissues [71]. Recently, more and more studies on the bile acid-induced IM via activating FXR have been reported (Table 1).

### TGR5

The bile acid-activated membrane receptor TGR5, widely distributed in the liver, intestine, brown adipose tissue, and immune cells, involves in the regulation of energy metabolism, glucose metabolism, and inflammatory response [72, 73]. Secondary bile acids LCA and DCA are potent agonists for TGR5 [74]. Zhen et al. revealed that DCA treatment induced HNF4α expression via TGR5 and following ERK1/2-pathway activation [42]. Thus, inhibition of the TGR5–HNF4α signaling-cascade response may be a potential therapeutic target to prevent the progression of the Correa cascade and the proceeding of GC. At present, further studies are needed regarding the role played by the bile acid receptor TGR5 in gastric intestinal metaplasia.

## Monitor and reverse gastric IM

### Monitor the gastric IM

Gastric IM, a key point in the multistep process of GC, is considered to be an important gastric premalignant lesion. Therefore, endoscopic surveillance of the progression of gastric IM is an effective means of prevention of GC. Operative link for gastric cancer metaplasia assessment (OLGIM) indicates that OLGIM stage III and IV are significantly more likely to develop intraepithelial neoplasia than OLGIM stage 0–II [75]. It is recommended that the patients with OLGIM stage III/IV should be followed up with a high-quality endoscopy every 3 years [76]. American Gastroenterological Association recommended that the patients with incidental findings of gastric IM on endoscopy should detect and eradicate H. pylori. Endoscopic surveillance is reasonable for gastric IM patients at high risk of GC, including those with incomplete IM, extensive IM involving the gastric body and sinuses or the gastric horn, and a family history of GC. Routine short-interval repeat endoscopy for the purpose of risk stratification is not recommended for patients with gastric IM [77]. Meanwhile, the application of pigment endoscopy and narrow-band imaging endoscopy improves the detection rate of gastric IM [78, 79]. The confocal laser endomicroscopy has a sensitivity and specificity of 98.13% and 95.33% for the diagnosis of IM, and can also differentiate its subtypes [80].

Long-term exposure to bile acids has been shown to increase the risk of transition from normal mucosa to IM, and eventually leads to the development of GC over many years [81–83]. However, there are no clear studies on whether bile-reflux monitoring can be used to monitor and delay the development of gastric IM. Combining our previous description of bile acid and gastric IM, we may speculate that bile- reflux monitoring will be a possible means for monitoring and delaying the development of gastric IM in the future. Endoscopy may show duodenogastric reflux or the presence of bile in the stomach. Endoscopic findings most often include bile pooling, erythema of the gastric mucosa, thickened gastric folds, erosions, and gastric atrophy [84]. Histologic features include foveolar hyperplasia, edema, acute or chronic inflammation, and IM [84]. These features are not specific to bile reflux, which are similar to those found in some chemical injuries, so it is important to exclude competing etiologies. The aspiration of gastric juice under endoscopy allows chemical analysis of the fluid and determines the presence of bile acids [85]. Modalities for establishing bile reflux also include measurement of bilirubin in the stomach using a fiber-optic spectrophtometer, or biliary radionuclide scanning showing radiotracer in the stomach [86, 87].

### The reversal of gastric IM

As a general treatment approach to bile reflux, the first step may be to stop any food or nonessential medications that might cause gastrointestinal motor dysfunction, such as strong tea, coffee, alcohol, and opioids [88]. Proton-pump inhibitors are common, which reduce the secretion of gastric acid and duodenal contents [89]. In addition, it also has a potent anti-inflammatory effect via inhibition of chemokines and adhesion molecules [90, 91]. Gastric mucosal-protective agents with the binding ability of bile acids, such as hydrotalcite, strengthen the gastric mucosal barrier, thereby alleviating gastric mucosal injury caused by bile reflux [92]. Prokinetic agents, such as mosapride and domperidone, can reduce bile reflux [93, 94]. In addition, short-term use of ursodeoxycholic acid (UDCA) may be appropriate. It changes the relative concentrations of lipophilic bile acids with high cytotoxicity, and with time promotes mucosal healing [95, 96]. Combination therapy could be tried if individual therapies are ineffective. Surgical management of bile reflux may be considered in severely symptomatic patients, especially those with reflux caused by previous surgery. The most commonly used operative procedures include the Roux-en-Y procedure, the Braun enteroenterostomy, and Henley jejunal interposition. A Roux-en-Y choledochojejunostomy can be used to divert bile directly from the biliary tree after cholecystectomy [97]. In addition, surgical treatment is also suitable for patients with cancer or poor drug-treatment outcomes. In summary, the symptoms caused by bile reflux can be alleviated by drugs and surgery. However, there are no high-quality studies on whether the treatment of bile reflux can reverse IM.

Currently, there is a controversial debate whether or not gastric IM is reversible. It is generally believed that H. pylori eradication before the occurrence of gastric IM can help control gastritis, while once gastric IM is established, H. pylori eradication cannot reverse gastric IM but can help prevent or delay the progression of gastric IM [98, 99]. However, a meta-analysis showed that H. pylori eradication did not reverse gastric IM and reduce the risk of GC in patients with IM [100]. Therefore, more evidence is needed to determine whether H. pylori eradication can delay and reverse IM. In addition, a clinical study showed that a healthy diet may play a key role in inhibiting IM, which can be used as a primary prevention measure for the disease [101]. Certain vitamins and selenium may reduce the risk of GC [102–104]. For some patients with low folic acid level, appropriate supplementation of folic acid can alleviate progress of precancerous lesions and reduce the incidence of GC [105, 106]. Other studies have shown that endoscopic radiofrequency ablation provides a new direction for delaying gastric mucosal atrophy and IM, but more clinical evidence is still needed. In recent years, traditional Chinese medicine (TCM) in the treatment of gastric IM has highlighted obvious advantages. However, the lack of standardized TCM-syndrome diagnosis and the lack of clinical evidence to support need to be addressed in subsequent studies. It has been reported that resveratrol has a potential reversal effect on bile acid-induced gastric IM via PI3K/AKT/P-FoxO4 signaling pathway [107]. However, more studies are needed to further investigate whether the mechanism of bile reflux can be targeted to reverse IM.

## Summary and prospects

**Fig. 3:**
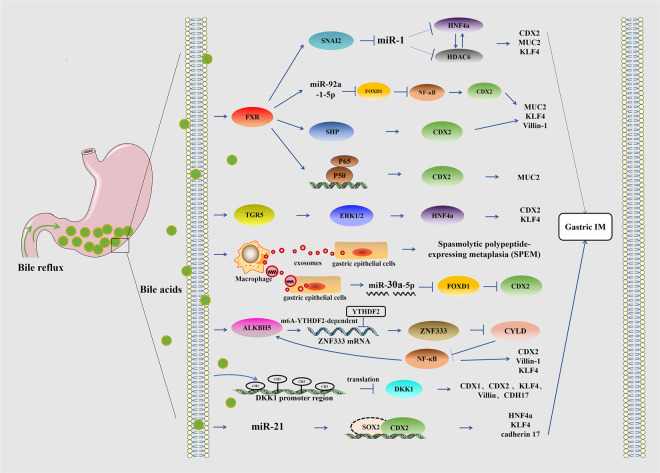
Mechanism of bile acid-induced gastric IM. Bile acids increase intestinal marker expression via the pathways of FXR/SNAI2/miR-1, FXR/miR-92a-1–5p/FOXD1/NF-κB/CDX2, FXR/SHP/CDX2, FXR/NF-κB/CDX2, TGR5/ERK1/2/HNF4a, miR-21/SOX2/CDX2, and the promoter methylation and downregulation of DKK1 in the stomach. An m6A modification-associated positive feedforward loop between ALKBH5 and NF-kB signaling is involved in generating the IM. Bile acid-induced ALKBH5 enhances ZNF333 levels through an m6A–YTHDF2-dependent manner. In addition, macrophage-derived exosomes facilitate cellular communication between macrophages and gastric epithelial cells in the DCA microenvironment, which promotes the development of SPEM and contributes to gastric IM by transferring miR-30a-5p.

Gastric IM, an important precancerous lesion of GC, is considered a critical stage in the prevention and control of GC because it provides a wide time window for clinical intervention of GC. Early diagnosis and treatment of gastric IM are of great significance for the prevention of GC. As shown in Fig. 3, bile reflux into the stomach acts as a signaling molecule involved in the development of IM by regulating downstream signaling pathways. Currently, there are few mechanisms for bile acid-induced gastric IM, including activation of bile acid receptors of FXR and TGR5, secretion of exosomes by macrophages acting on gastric epithelial cells, epigenetics, and miRNA involvement, which ultimately lead to the elevation of IM biomarkers. At present, some achievements have been made on the classification, pathogenic factors, and prevention and treatment methods of gastric IM. However, the molecular regulation mechanism of bile acid-induced gastric IM has not been fully clarified, and the related process cannot be fully explained. Therefore, more studies are needed to explore the mechanism of gastric IM and clarify the specific mechanism of intestinal metaplasia induced by bile acid reflux to provide a new plan for the prevention and treatment of early GC.

## Data Availability

Data sharing is not applicable to this article, as no data sets were generated or analyzed during the current study.

## References

[1] Sung H, Ferlay J, Siegel RL, Laversanne M, Soerjomataram I, Jemal A (2021). Global cancer statistics 2020: GLOBOCAN estimates of incidence and mortality worldwide for 36 cancers in 185 countries. CA Cancer J Clin.

[2] Allemani C, Matsuda T, Di Carlo V, Harewood R, Matz M, Nikšić M (2018). Global surveillance of trends in cancer survival 2000–14 (CONCORD-3): analysis of individual records for 37 513 025 patients diagnosed with one of 18 cancers from 322 population-based registries in 71 countries. Lancet.

[3] Correa P (1988). A human model of gastric carcinogenesis. Cancer Res.

[4] Shimoyama T, Fukuda S, Tanaka M, Nakaji S, Munakata A (2000). Evaluation of the applicability of the gastric carcinoma risk index for intestinal type cancer in Japanese patients infected with Helicobacter pylori. Virchows Arch.

[5] Filipe MI, Muñoz N, Matko I, Kato I, Pompe-Kirn V, Jutersek A (1994). Intestinal metaplasia types and the risk of gastric cancer: a cohort study in Slovenia. Int J Cancer.

[6] Straub D, Oude Elferink R, Jansen P, Bergman J, Parikh K, Krishnadath KK (2019). Glyco-conjugated bile acids drive the initial metaplastic gland formation from multi-layered glands through crypt-fission in a murine model. PLoS ONE.

[7] Shah SC, Gupta S, Li D, Morgan D, Mustafa RA, Gawron AJ (2020). Spotlight: gastric Intestinal Metaplasia. Gastroenterology.

[8] Alpízar-Alpízar W, Skindersoe ME, Rasmussen L, Kriegbaum MC, Christensen IJ, Lund IK, et al. *Helicobacter pylori* colonization drives urokinase receptor (uPAR) expression in murine gastric epithelium during early pathogenesis. Microorganisms. 2020;8:1019. 10.3390/microorganisms8071019 PMC7409347 32660136

[9] Joo MK, Park JJ, Chun HJ (2019). Proton pump inhibitor: the dual role in gastric cancer. World J Gastroenterol.

[10] Zhang LY, Zhang J, Li D, Liu Y, Zhang DL, Liu CF (2021). Bile reflux is an independent risk factor for precancerous gastric lesions and gastric cancer: an observational cross-sectional study. J Dig Dis.

[11] Shah SC, Gawron AJ, Mustafa RA, Piazuelo MB (2020). Histologic subtyping of gastric intestinal metaplasia: overview and considerations for clinical practice. Gastroenterology.

[12] Jass JR, Filipe MI (1981). The mucin profiles of normal gastric mucosa, intestinal metaplasia and its variants and gastric carcinoma. Histochem J.

[13] Shao L, Li P, Ye J, Chen J, Han Y, Cai J (2018). Risk of gastric cancer among patients with gastric intestinal metaplasia. Int J Cancer.

[14] Busuttil RA, Boussioutas A (2009). Intestinal metaplasia: a premalignant lesion involved in gastric carcinogenesis. J Gastroenterol Hepatol.

[15] Shichijo S, Hirata Y, Niikura R, Hayakawa Y, Yamada A, Ushiku T (2016). Histologic intestinal metaplasia and endoscopic atrophy are predictors of gastric cancer development after Helicobacter pylori eradication. Gastrointest Endosc.

[16] Sakitani K, Hirata Y, Watabe H, Yamada A, Sugimoto T, Yamaji Y (2011). Gastric cancer risk according to the distribution of intestinal metaplasia and neutrophil infiltration. J Gastroenterol Hepatol.

[17] Liu H, Hu C, Zhang X, Jia W (2018). Role of gut microbiota, bile acids and their cross-talk in the effects of bariatric surgery on obesity and type 2 diabetes. J Diabetes Investig.

[18] Stellaard F, Sackmann M, Sauerbruch T, Paumgartner G (1984). Simultaneous determination of cholic acid and chenodeoxycholic acid pool sizes and fractional turnover rates in human serum using 13C-labeled bile acids. J Lipid Res.

[19] Zwartjes M, Gerdes V, Nieuwdorp M. The role of gut microbiota and its produced metabolites in obesity, dyslipidemia, adipocyte dysfunction, and its interventions. Metabolites. 2021;11:531. 10.3390/metabo11080531 PMC8398981 34436472

[20] Vivian D, Polli JE (2014). Synthesis and in vitro evaluation of bile acid prodrugs of floxuridine to target the liver. Int J Pharm.

[21] Li D, Zhang J, Yao WZ, Zhang DL, Feng CC, He Q (2020). The relationship between gastric cancer, its precancerous lesions and bile reflux: a retrospective study. J Dig Dis.

[22] Graham SF, Rey NL, Ugur Z, Yilmaz A, Sherman E, Maddens M, et al. Metabolomic profiling of bile acids in an experimental model of prodromal Parkinson’s disease. Metabolites. 2018;8:71. 10.3390/metabo8040071 PMC6316593 30384419

[23] Bechi P, Amorosi A, Mazzanti R, Dei R, Bianchi S, Mugnai L (1993). Reflux-related gastric mucosal injury is associated with increased mucosal histamine content in humans. Gastroenterology.

[24] Goldman A, Shahidullah M, Goldman D, Khailova L, Watts G, Delamere N (2010). A novel mechanism of acid and bile acid-induced DNA damage involving Na+/H+ exchanger: implication for Barrett’s oesophagus. Gut.

[25] Choi J, Kim SG, Yoon H, Im JP, Kim JS, Kim WH (2014). Eradication of Helicobacter pylori after endoscopic resection of gastric tumors does not reduce incidence of metachronous gastric carcinoma. Clin Gastroenterol Hepatol.

[26] Igarashi M, Nakae H, Matsuoka T, Takahashi S, Hisada T, Tomita J (2017). Alteration in the gastric microbiota and its restoration by probiotics in patients with functional dyspepsia. BMJ Open Gastroenterol.

[27] Takahashi Y, Uno K, Iijima K, Abe Y, Koike T, Asano N (2018). Acidic bile salts induces mucosal barrier dysfunction through let-7a reduction during gastric carcinogenesis after *Helicobacter pylori* eradication. Oncotarget.

[28] Matsuhisa T, Arakawa T, Watanabe T, Tokutomi T, Sakurai K, Okamura S (2013). Relation between bile acid reflux into the stomach and the risk of atrophic gastritis and intestinal metaplasia: a multicenter study of 2283 cases. Dig Endosc.

[29] Tatsugami M, Ito M, Tanaka S, Yoshihara M, Matsui H, Haruma K (2012). Bile acid promotes intestinal metaplasia and gastric carcinogenesis. Cancer Epidemiol Biomark Prev.

[30] Mutoh H, Hayakawa H, Sakamoto H, Sashikawa M, Sugano K (2009). Transgenic Cdx2 induces endogenous Cdx1 in intestinal metaplasia of Cdx2-transgenic mouse stomach. FEBS J.

[31] di Pietro M, Lao-Sirieix P, Boyle S, Cassidy A, Castillo D, Saadi A (2012). Evidence for a functional role of epigenetically regulated midcluster HOXB genes in the development of Barrett esophagus. Proc Natl Acad Sci USA.

[32] Raghoebir L, Bakker ER, Mills JC, Swagemakers S, Kempen MB, Munck AB (2012). SOX2 redirects the developmental fate of the intestinal epithelium toward a premature gastric phenotype. J Mol Cell Biol.

[33] Park ET, Gum JR, Kakar S, Kwon SW, Deng G, Kim YS (2008). Aberrant expression of SOX2 upregulates MUC5AC gastric foveolar mucin in mucinous cancers of the colorectum and related lesions. Int J Cancer.

[34] Bornschein J, Tóth K, Selgrad M, Kuester D, Wex T, Molnár B (2013). Dysregulation of CDX1, CDX2 and SOX2 in patients with gastric cancer also affects the non-malignant mucosa. J Clin Pathol.

[35] Tsukamoto T, Inada K, Tanaka H, Mizoshita T, Mihara M, Ushijima T (2004). Down-regulation of a gastric transcription factor, Sox2, and ectopic expression of intestinal homeobox genes, Cdx1 and Cdx2: inverse correlation during progression from gastric/intestinal-mixed to complete intestinal metaplasia. J Cancer Res Clin Oncol.

[36] Raghoebir L, Biermann K, Buscop-van Kempen M, Wijnen RM, Tibboel D, Smits R (2013). Disturbed balance between SOX2 and CDX2 in human vitelline duct anomalies and intestinal duplications. Virchows Arch.

[37] Yuan T, Ni Z, Han C, Min Y, Sun N, Liu C (2019). SOX2 interferes with the function of CDX2 in bile acid-induced gastric intestinal metaplasia. Cancer Cell Int.

[38] Babeu JP, Boudreau F (2014). Hepatocyte nuclear factor 4-alpha involvement in liver and intestinal inflammatory networks. World J Gastroenterol.

[39] Yin L, Ma H, Ge X, Edwards PA, Zhang Y (2011). Hepatic hepatocyte nuclear factor 4α is essential for maintaining triglyceride and cholesterol homeostasis. Arterioscler Thromb Vasc Biol.

[40] Bagwell AM, Bailly A, Mychaleckyj JC, Freedman BI, Bowden DW (2004). Comparative genomic analysis of the HNF-4alpha transcription factor gene. Mol Genet Metab.

[41] Freund JN, Duluc I, Reimund JM, Gross I, Domon-Dell C (2015). Extending the functions of the homeotic transcription factor Cdx2 in the digestive system through nontranscriptional activities. World J Gastroenterol.

[42] Ni Z, Min Y, Han C, Yuan T, Lu W, Ashktorab H (2020). TGR5-HNF4α axis contributes to bile acid-induced gastric intestinal metaplasia markers expression. Cell Death Disco.

[43] Wang N, Chen M, Ni Z, Li T, Zeng J, Lu G (2021). HDAC6/HNF4α loop mediated by miR-1 promotes bile acids-induced gastric intestinal metaplasia. Gastric Cancer.

[44] Kato M, Slack FJ (2008). microRNAs: small molecules with big roles - C. elegans to human cancer. Biol Cell.

[45] Lynam-Lennon N, Maher SG, Reynolds JV (2009). The roles of microRNA in cancer and apoptosis. Biol Rev Camb Philos Soc.

[46] Mutoh H, Sashikawa M, Sugano K (2011). Sox2 expression is maintained while gastric phenotype is completely lost in Cdx2-induced intestinal metaplastic mucosa. Differentiation.

[47] Li T, Guo H, Li H, Jiang Y, Zhuang K, Lei C (2019). MicroRNA-92a-1-5p increases CDX2 by targeting FOXD1 in bile acids-induced gastric intestinal metaplasia. Gut.

[48] Sahebi R, Langari H, Fathinezhad Z, Bahari Sani Z, Avan A, Ghayour Mobarhan M (2020). Exosomes: New insights into cancer mechanisms. J Cell Biochem.

[49] Gowda R, Robertson BM, Iyer S, Barry J, Dinavahi SS, Robertson GP (2020). The role of exosomes in metastasis and progression of melanoma. Cancer Treat Rev.

[50] Taylor DD, Gercel-Taylor C (2008). MicroRNA signatures of tumor-derived exosomes as diagnostic biomarkers of ovarian cancer. Gynecol Oncol.

[51] Xu X, Cheng J, Luo S, Huang D, Xu J, Qian Y (2020). Deoxycholic acid-stimulated macrophage-derived exosomes promote intestinal metaplasia and suppress proliferation in human gastric epithelial cells. Eur J Pharm.

[52] Wu H, Fu M, Liu J, Chong W, Fang Z, Du F (2021). The role and application of small extracellular vesicles in gastric cancer. Mol Cancer.

[53] Xu X, Cheng J, Luo S, Gong X, Huang D, Xu J (2020). Deoxycholic acid-stimulated macrophage-derived exosomes promote spasmolytic polypeptide-expressing metaplasia in the stomach. Biochem Biophys Res Commun.

[54] Zhou J, Wan J, Shu XE, Mao Y, Liu XM, Yuan X (2018). N6-Methyladenosine Guides mRNA alternative translation during integrated stress response. Mol. Cell.

[55] Zhang C, Samanta D, Lu H, Bullen JW, Zhang H, Chen I (2016). Hypoxia induces the breast cancer stem cell phenotype by HIF-dependent and ALKBH5-mediated m^6^A-demethylation of NANOG mRNA. Proc Natl Acad Sci USA.

[56] Yue B, Cui R, Zheng R, Jin W, Song C, Bao T (2021). Essential role of ALKBH5-mediated RNA demethylation modification in bile acid-induced gastric intestinal metaplasia. Mol Ther Nucleic Acids.

[57] Steinhart Z, Angers S. Wnt signaling in development and tissue homeostasis. Development. 2018;145:dev146589. 10.1242/dev.146589 29884654

[58] Glinka A, Wu W, Delius H, Monaghan AP, Blumenstock C, Niehrs C (1998). Dickkopf-1 is a member of a new family of secreted proteins and functions in head induction. Nature.

[59] Niehrs C (2006). Function and biological roles of the Dickkopf family of Wnt modulators. Oncogene.

[60] Lu W, Ni Z, Tong M, Jiang S, Zhang J, Feng C (2020). DKK1 is epigenetically downregulated by promoter methylation and inhibits bile acid-induced gastric intestinal metaplasia. Biochem Biophys Res Commun.

[61] Ticho AL, Malhotra P, Dudeja PK, Gill RK, Alrefai WA (2019). Bile acid receptors and gastrointestinal functions. Liver Res.

[62] Chávez-Talavera O, Tailleux A, Lefebvre P, Staels B (2017). Bile acid control of metabolism and inflammation in obesity, type 2 diabetes, dyslipidemia, and nonalcoholic fatty liver disease. Gastroenterology.

[63] Donkers JM, Roscam Abbing R, van de Graaf S (2019). Developments in bile salt based therapies: a critical overview. Biochem Pharm.

[64] Jia W, Xie G, Jia W (2018). Bile acid-microbiota crosstalk in gastrointestinal inflammation and carcinogenesis. Nat Rev Gastroenterol Hepatol.

[65] Vaquero J, Monte MJ, Dominguez M, Muntané J, Marin JJ (2013). Differential activation of the human farnesoid X receptor depends on the pattern of expressed isoforms and the bile acid pool composition. Biochem Pharm.

[66] Song P, Rockwell CE, Cui JY, Klaassen CD (2015). Individual bile acids have differential effects on bile acid signaling in mice. Toxicol Appl Pharm.

[67] Beuers U, Trauner M, Jansen P, Poupon R (2015). New paradigms in the treatment of hepatic cholestasis: from UDCA to FXR, PXR and beyond. J Hepatol.

[68] Ku HJ, Kim HY, Kim HH, Park HJ, Cheong JH (2014). Bile acid increases expression of the histamine-producing enzyme, histidine decarboxylase, in gastric cells. World J Gastroenterol.

[69] Xu Y, Watanabe T, Tanigawa T, Machida H, Okazaki H, Yamagami H (2010). Bile acids induce cdx2 expression through the farnesoid x receptor in gastric epithelial cells. J Clin Biochem Nutr.

[70] Li S, Chen X, Zhou L, Wang BM (2015). Farnesoid X receptor signal is involved in deoxycholic acid-induced intestinal metaplasia of normal human gastric epithelial cells. Oncol Rep.

[71] Zhou H, Ni Z, Li T, Su L, Zhang L, Liu N (2018). Activation of FXR promotes intestinal metaplasia of gastric cells via SHP-dependent upregulation of the expression of CDX2. Oncol Lett.

[72] Arab JP, Karpen SJ, Dawson PA, Arrese M, Trauner M (2017). Bile acids and nonalcoholic fatty liver disease: Molecular insights and therapeutic perspectives. Hepatology.

[73] Martinot E, Sèdes L, Baptissart M, Lobaccaro JM, Caira F, Beaudoin C (2017). Bile acids and their receptors. Mol Asp Med.

[74] Kawamata Y, Fujii R, Hosoya M, Harada M, Yoshida H, Miwa M (2003). A G protein-coupled receptor responsive to bile acids. J Biol Chem.

[75] Rugge M, Fassan M, Pizzi M, Farinati F, Sturniolo GC, Plebani M (2011). Operative link for gastritis assessment vs operative link on intestinal metaplasia assessment. World J Gastroenterol.

[76] Pimentel-Nunes P, Libânio D, Marcos-Pinto R, Areia M, Leja M, Esposito G (2019). Management of epithelial precancerous conditions and lesions in the stomach (MAPS II): European Society of Gastrointestinal Endoscopy (ESGE), European Helicobacter and Microbiota Study Group (EHMSG), European Society of Pathology (ESP), and Sociedade Portuguesa de Endoscopia Digestiva (SPED) guideline update 2019. Endoscopy.

[77] Gupta S, Li D, El Serag HB, Davitkov P, Altayar O, Sultan S (2020). AGA clinical practice guidelines on management of gastric intestinal metaplasia. Gastroenterology.

[78] Capelle LG, Haringsma J, de Vries AC, Steyerberg EW, Biermann K, van Dekken H (2010). Narrow band imaging for the detection of gastric intestinal metaplasia and dysplasia during surveillance endoscopy. Dig Dis Sci.

[79] Calle Astudillo G, Jerves T, Pesántez L, Calle P, Gutiérrez A, Calle G (2013). [Utility of routine gastric biopsies and staining with methylene blue in the diagnosis of intestinal metaplasia in patients over 40 years]. Acta Gastroenterol Latinoam.

[80] Guo YT, Li YQ, Yu T, Zhang TG, Zhang JN, Liu H (2008). Diagnosis of gastric intestinal metaplasia with confocal laser endomicroscopy in vivo: a prospective study. Endoscopy.

[81] Cao W, Tian W, Hong J, Li D, Tavares R, Noble L (2013). Expression of bile acid receptor TGR5 in gastric adenocarcinoma. Am J Physiol Gastrointest Liver Physiol.

[82] Camilo V, Garrido M, Valente P, Ricardo S, Amaral AL, Barros R (2015). Differentiation reprogramming in gastric intestinal metaplasia and dysplasia: role of SOX2 and CDX2. Histopathology.

[83] Niu H, Jia Y, Li T, Su B (2017). SOX2 inhibition promotes promoter demethylation of CDX2 to facilitate gastric intestinal metaplasia. Dig Dis Sci.

[84] Vere CC, Cazacu S, Comănescu V, Mogoantă L, Rogoveanu I, Ciurea T (2005). Endoscopical and histological features in bile reflux gastritis. Rom J Morphol Embryol.

[85] Mi S, Lim DW, Turner JM, Wales PW, Curtis JM (2016). Determination of bile acids in piglet bile by solid phase extraction and liquid chromatography-electrospray tandem mass spectrometry. Lipids.

[86] Gerard PS, Gerczuk P, Finestone H (2007). Bile reflux in the esophagus demonstrated by HIDA scintigraphy. Clin Nucl Med.

[87] Kuran S, Parlak E, Aydog G, Kacar S, Sasmaz N, Ozden A (2008). Bile reflux index after therapeutic biliary procedures. BMC Gastroenterol.

[88] Thapa N, Kappus M, Hurt R, Diamond S (2019). Implications of the opioid epidemic for the clinical gastroenterology practice. Curr Gastroenterol Rep.

[89] Vinter-Jensen L, Kraglund K, Pedersen SA (1989). A double-blind placebo-controlled trial of omeprazole on characteristics of the migrating motor complex in healthy volunteers. Aliment Pharm Ther.

[90] Handa O, Yoshida N, Fujita N, Tanaka Y, Ueda M, Takagi T (2006). Molecular mechanisms involved in anti-inflammatory effects of proton pump inhibitors. Inflamm Res.

[91] Chen H, Li X, Ge Z, Gao Y, Chen X, Cui Y (2010). Rabeprazole combined with hydrotalcite is effective for patients with bile reflux gastritis after cholecystectomy. Can J Gastroenterol.

[92] Huo X, Zhang X, Yu C, Zhang Q, Cheng E, Wang DH (2014). In oesophageal squamous cells exposed to acidic bile salt medium, omeprazole inhibits IL-8 expression through effects on nuclear factor-κB and activator protein-1. Gut.

[93] Zhang CX, Qin YM, Guo BR (2010). Clinical study on the treatment of gastroesophageal reflux by acupuncture. Chin J Integr Med.

[94] Chen SL, Ji JR, Xu P, Cao ZJ, Mo JZ, Fang JY (2010). Effect of domperidone therapy on nocturnal dyspeptic symptoms of functional dyspepsia patients. World J Gastroenterol.

[95] Stefaniwsky AB, Tint GS, Speck J, Shefer S, Salen G (1985). Ursodeoxycholic acid treatment of bile reflux gastritis. Gastroenterology.

[96] Shi J, Li Z, Zeng X, Lin Y, Xie WF (2009). Ursodeoxycholic acid in primary sclerosing cholangitis: meta-analysis of randomized controlled trials. Hepatol Res.

[97] Madura JA (2003). Primary bile reflux gastritis: diagnosis and surgical treatment. Am J Surg.

[98] Lee YC, Chen TH, Chiu HM, Shun CT, Chiang H, Liu TY (2013). The benefit of mass eradication of Helicobacter pylori infection: a community-based study of gastric cancer prevention. Gut.

[99] Liu KS, Wong IO, Leung WK (2016). Helicobacter pylori associated gastric intestinal metaplasia: Treatment and surveillance. World J Gastroenterol.

[100] Chen HN, Wang Z, Li X, Zhou ZG (2016). Helicobacter pylori eradication cannot reduce the risk of gastric cancer in patients with intestinal metaplasia and dysplasia: evidence from a meta-analysis. Gastric Cancer.

[101] Oh S, Kim N, Yoon H, Choi YJ, Lee JY, Park KJ (2013). Risk factors of atrophic gastritis and intestinal metaplasia in first-degree relatives of gastric cancer patients compared with age-sex matched controls. J Cancer Prev.

[102] Ma JL, Zhang L, Brown LM, Li JY, Shen L, Pan KF (2012). Fifteen-year effects of Helicobacter pylori, garlic, and vitamin treatments on gastric cancer incidence and mortality. J Natl Cancer Inst.

[103] Yuan JM, Ross RK, Gao YT, Qu YH, Chu XD, Yu MC (2004). Prediagnostic levels of serum micronutrients in relation to risk of gastric cancer in Shanghai, China. Cancer Epidemiol Biomark Prev.

[104] van den Brandt PA, Goldbohm RA (2006). Nutrition in the prevention of gastrointestinal cancer. Best Pr Res Clin Gastroenterol.

[105] Fang JY, Xiao SD (2003). Folic acid, polymorphism of methyl-group metabolism genes, and DNA methylation in relation to GI carcinogenesis. J Gastroenterol.

[106] Xiao SD, Meng XJ, Shi Y, Hu YB, Zhu SS, Wang CW (2002). Interventional study of high dose folic acid in gastric carcinogenesis in beagles. Gut.

[107] Lu W, Ni Z, Jiang S, Tong M, Zhang J, Zhao J (2021). Resveratrol inhibits bile acid-induced gastric intestinal metaplasia via the PI3K/AKT/p-FoxO4 signalling pathway. Phytother Res.

[108] Yu JH, Zheng JB, Qi J, Yang K, Wu YH, Wang K (2019). Bile acids promote gastric intestinal metaplasia by upregulating CDX2 and MUC2 expression via the FXR/NF-κB signalling pathway. Int J Oncol.

[109] Wang N, Wu S, Zhao J, Chen M, Zeng J, Lu G (2021). Bile acids increase intestinal marker expression via the FXR/SNAI2/miR-1 axis in the stomach. Cell Oncol.

